# Event-related potential studies of post-traumatic stress disorder: a critical review and synthesis

**DOI:** 10.1186/2045-5380-1-5

**Published:** 2011-10-12

**Authors:** Arash Javanbakht, Israel Liberzon, Alireza Amirsadri, Klevest Gjini, Nash N Boutros

**Affiliations:** 1 Department of Psychiatry, University of Michigan, Ann Arbor, 1500 East Medical Center Drive, Ann Arbor, MI 48109, USA; 2 Department of Psychiatry and Behavioral Neurosciences, Wayne State University, 540 E Canfield, Detroit, MI 48201-1998, USA

## Abstract

Despite the sparseness of the currently available data, there is accumulating evidence of information processing impairment in post-traumatic stress disorder (PTSD). Studies of event-related potentials (ERPs) are the main tool in real time examination of information processing. In this paper, we sought to critically review the ERP evidence of information processing abnormalities in patients with PTSD. We also examined the evidence supporting the existence of a relationship between ERP abnormalities and symptom profiles or severity in PTSD patients. An extensive Medline search was performed. Keywords included PTSD or post-traumatic stress disorder, electrophysiology or EEG, electrophysiology, P50, P100, N100, P2, P200, P3, P300, sensory gating, CNV (contingent negative variation) and MMN (mismatch negativity). We limited the review to ERP adult human studies with control groups which were reported in the English language. After applying our inclusion-exclusion review criteria, 36 studies were included. Subjects exposed to wide ranges of military and civilian traumas were studied in these reports. Presented stimuli were both auditory and visual. The most widely studied components included P300, P50 gating, N100 and P200. Most of the studies reported increased P300 response to trauma-related stimuli in PTSD patients. A smaller group of studies reported dampening of responses or no change in responses to trauma-related and/or unrelated stimuli. P50 studies were strongly suggestive of impaired gating in patients with PTSD. In conclusion, the majority of reports support evidence of information processing abnormalities in patients with PTSD diagnosis. The predominance of evidence suggests presence of mid-latency and late ERP components differences in PTSD patients in comparison to healthy controls. Heterogeneity of assessment methods used contributes to difficulties in reaching firm conclusions regarding the nature of these differences. We suggest that future ERP-PTSD studies utilize standardized assessment scales that provide detailed information regarding the symptom clusters and the degree of symptom severity. This would allow assessment of electrophysiological indices-clinical symptoms relationships. Based on the available data, we suggest that ERP abnormalities in PTSD are possibly affected by the level of illness severity. If supported by future research, ERP studies may be used for both initial assessment and treatment follow-up.

## Introduction

Although post-traumatic stress disorder (PTSD) is classified as an 'anxiety disorder', evidence of cognitive and information processing (IP) abnormalities in PTSD has been accumulating [1]. While many studies on emotional processing abnormalities in PTSD exist, event-related potentials (ERPs) studies focusing on early stages of IP abnormalities in PTSD are limited in number. The aim of this review was to summarize ERP findings in PTSD and determine whether there are consistent patterns of IP deviations reported in this disorder. We also sought to gain possible insight into clinical correlates of these differences. Another aim of this review was to assess if we could present suggestions for future research methods.

### Event-related brain potentials and components

Brain ERPs are the main tools available for clinical investigators to probe IP in real time, as they can assess different phases of IP in the human brain [2]. Abnormality of the initial phase of IP (the 0 to 20 ms following auditory or visual stimulation), where information is conducted through subcortical structures on its way to the cerebral cortex, is usually linked to brain stem abnormalities [3]. Abnormalities at this stage of IP are rarely reported in psychiatric patients [4]. Due to the extremely small number of ERP studies examining this stage of IP in association with PTSD, this IP stage is not further discussed in this report.

The midlatency range of information processing (following the early stage and spanning 20 to 200 ms following stimulation), when signal registration and filtering out (gating) of redundant information takes place [5], has been shown to be abnormal in a large number of psychiatric and neuropsychiatric conditions [6]. Auditory midlatency range is represented by three major event-related response components: the P50 (40 to 80 ms), N100 (75 to 150 ms) and the P200 (150 to 250 ms) [7]. Two variables are routinely examined in association with all ERPs: amplitude (how large the response is) and latency (how long after the stimulus the response is maximally seen). Amplitudes and latencies are examined using trains of identical stimuli and averaging the resulting responses [8]. Midlatency ERP responses are also widely used to examine habituation (or sensory gating) in the brain [9]. A standard paired-stimulus paradigm (S1-S2) is used for the purpose of examining habituation or gating of the P50/N100/P200 components with stimulus repetition. Habituation or gating is routinely assessed as the ratio of the responses to S2 stimuli as compared to responses to S1 stimuli (S2/S1 × 100). Higher ratios reflect decreased gating ability [10]. All three midlatency auditory event-related response (MLAER) components are demonstrated to have decreased gating in association with psychosis [5].

The later stage of IP is when higher cognitive manipulations occur [11]. The P300 ERP component is a large positive electroencephalography (EEG) deflection elicited approximately 300 ms after an individual detects a deviant stimulus imbedded among ongoing repeating stimuli [11]. The amplitude of the P300 has been linked to the amount of attentional resources allocated to the experimental task. P300 latency has been linked to the speed of IP. P500 is a positive deflection which appears between 300 and 900 ms after stimulus presentation and is believed to be involved in updating working memory representations of a specified stimulus [12]. Abnormalities of these ERP, especially the P300 (250 to 350 ms), are common in psychiatric populations.

## Methods

A detailed Medline search was performed. Keywords included PTSD or post-traumatic stress disorder and EEG, electrophysiology, P50, P100, N100, P2, P200, P3, P300, sensory gating, CNV (contingent negative variation), and MMN (mismatch negativity). The search was limited to human studies reported in the English language. Because of the very small number of electrophysiological studies in children with mental illnesses in general, three of the manuscripts which reported children studies were not included in this review. Papers which were solely EEG or sleep studies in PTSD without inclusion of ERPs were also not included. We considered only studies which included a healthy control group, enabling extraction of the differences between ERP responses in patients with PTSD and those of healthy participants. We then reviewed full texts of the selected manuscripts and summarized ERP responses from these studies in separate tables (Tables 1, 2, 3 and 4). We also extracted reported clinical correlates of the ERP results and summarized them in Table 5. This table shows the rating scales which were used in each study and the correlation of the scores of these tests with the ERP data.

**Table 1:** Studies examining the P50 and sensory gating

Study	Subjects	Paradigm	Results
[51]	46 prisoners of war 36 healthy controls	Checkerboard reversal (visual)	Significantly greater P50 amplitude and latency

[52]	10 inpatient combat PTSD 5 inpatient alcohol-dependents 5 combat-exposed and 5 combat-non-exposed healthy subjects	Paired click	Diminished P50 habituation in PTSD

[25]	13 female with sexual assault PTSD 16 healthy controls	Auditory oddball	No difference in P50 peak amplitude and latency

[53]	15 combat veterans 12 healthy control	Paired click	P50 amplitude in response to the conditioning stimulus did not differ. P50 T/C ratio was increased in PTSD subjects.

[54]	10 male veteran PTSD + 9 female rape victims matched control groups	Paired click	Decreased P50 gating

[32]	29 PTSD nurse veterans 38 non-PTSD	Paired click	Reduced P50 suppression associated with increased severity of general psychopathology, but not with PTSD.

[27]	12 urban violence PTSD/24 healthy subjects/12 schizophrenics	Paired click	Higher P50 ratios in subjects with PTSD

[55]	27 civilian with mixed types of trauma and 24 control subjects	Paired click	Impaired P50 suppression in PTSD subjects

[26]	Seven combat veterans with PTSD and 11 matched controls	Paired click	Impaired M50 gating in the right hemisphere in PTSD subjects. Thinner right STG (Superior Temporal Gyrus) cortical thickness was associated with worse right sensory gating in the PTSD group. The right S1 P50 source strength and gating ratio were correlated with PTSD symptomatology.

**Table 2:** Summary of the studies which included N100 component

Study	Subjects	Paradigm	Results
[31]	12 combat PTSD veterans 6 normal controls	Four tones intensity paradigm	No difference in N1 amplitude

[17]	20 Israeli combat veterans with PTSD 20 without PTSD	Visual oddball, trauma related non-related neutral stimuli	Combat-related pictures elicited enhanced N1 amplitude in PTSD group. Prolonged N1 latencies and reaction times to target stimuli in PTSD patients.

[56]	16 medicated, 9 un-medicated PTSD 10 healthy veterans	Auditory three-tone oddball	Longer N100 latencies in un-medicated PTSD patients compared to the medicated PTSD and healthy controls.

[51]	11 prisoners of war	Checkerboard reversal	Larger N75 amplitudes

[39]	11 PTSD survivors of a ship fire 9 psychiatric controls from the same ship	Auditory word and non-word oddball	Increased N1 latency to standard tones; Larger amplitude to emotionally meaningful words.

[25]	13 females with sexual assault PTSD 16 healthy controls	Auditory oddball	No difference in N100 amplitude and latency

[28]	17 civil PTSD 17 healthy controls	Auditory oddball	No difference in N100 amplitude or latency

[30]	36 civil PTSD 20 healthy 10 depressed 8 alcoholics	2000-Hz tone presented in increasing intensities	Increased N100 amplitudes

[38]	15 civil PTSD 15 controls	Visual presentation of angry alternating with neutral faces	Larger N110 to the angry compared to the neutral faces in the control group. Smaller and later N100 in PTSD subjects.

[35]	10 civil PTSD 10 controls	Auditory oddball	Larger N100 amplitude

[42]	19 PTSD 99 Alcohol dependence 16 personality disorder 25 anxiety or mood disorder	Visual presentation of happy, sad, and neutral faces	Larger N1 amplitudes to sad stimuli in frontotemporal leads in PTSD patients.

[57]	16 civil PTSD 16 schizophrenia 16 control subjects	Auditory Oddball	No difference in N1 amplitude and latency

[36]	14 PTSD [mixed etiologies] 12 controls	Auditory Oddball	No difference in N100 amplitude

**Table 3:** Summary of the studies which included P200 component

Study	Study Groups	Paradigm	Findings
[31]	12 combat PTSD veterans 6 normal controls	Four tones intensity paradigm	Reduced P2 amplitude intensity slope in PTSD subjects

[25]	13 female with sexual assault PTSD 16 healthy controls	Auditory oddball	Reduced amplitude and latency in response to deviant stimuli

[28]	17 civil PTSD 17 healthy controls	Auditory oddball	Smaller P200 to target and common tones. Earlier response to common but not target tones.

[30]	36 civil PTSD 20 healthy 10 depressed 8 alcoholics.	2000-Hz tone presented in intensity blocks of 65, 72.5, 80, 87.5, and 95 dB (SPL)	In normal subjects, depressed, and alcoholics, there was linear relationship between the tone intensity and P200 amplitude, which was not the case in combat related PTSD subjects.

[32]	29 PTSD nurse veterans 38 non-PTSD	Four-tone stimulus-intensity modulation paradigm	Increased P2 amplitude/intensity slope

[35]	10 civil PTSD 10 controls	Auditory oddball	No difference in P2 amplitude or latency

[29]	7 PTSD motor vehicle accident (MVA) 7 non-PTSD with MVA	Visual presentation of trauma related/unrelated/neutral pictures	Smaller P200 amplitude Larger response to trauma-related images in non-PTSD and healthy controls

[36]	14 PTSD [mixed etiologies] 12 controls	Auditory oddball	No difference in P200 amplitude

[33]	12 combat exposed veterans with PTSD and 33 without PTSD and their twins	Four-tone stimulus-intensity modulation paradigm	Increased P2 amplitude intensity slope in PTSD veterans. P2 amplitude intensity slopes were related to higher combat exposure, CAPS Total, and re-experience symptoms severity scores in the combat-exposed veterans but not to the remaining PTSD symptom cluster scores or the SCL-90-R (Symptom Checklist 90 Revised) general psychopathology, anxiety, or depression subscale scores. Higher combat-exposure scores, but not CAPS Total or subscale scores, were also related to increased P2 amplitude.

[34]	12 PTSD and 12 control survivors of earthquake	Subliminal visual presentation of earthquake-related/unrelated words	Increased P2 amplitude in the PTSD group in response to the trauma-related stimuli

**Table 4:** Summary of the studies which included P300 component

Study	Study Groups	Paradigm	Findings
[17]	20 Israeli combat veterans with PTSD 20 without PTSD	Modified target detection visual oddball paradigm, trauma related non-related neutral stimuli	Accentuated P300 amplitudes to target stimuli in both controls and PTSD patients Enhanced P300 amplitude in response to non-target combat related pictures in PTSD group Prolonged P300 latencies and reaction times to target stimuli were prolonged in PTSD subjects Increased latency in response to trauma-related stimuli in PTSD subjects

[50]	20 Israeli veterans with PTSD 20 without PTSD	Modified target detection visual oddball paradigm, trauma related, unrelated, neutral pictures	Smaller response to non-target images in the control, but equal responses to both target and non-target stimuli in the PTSD group No group difference for the target stimuli, but larger response to the non-target in the PTSD group. P300 could correctly classify 90% of PTSD and 90% of non-PTSD subjects. Increased latency of P300 response to combat-related images in relation with the severity of intrusive symptoms. This relation was negative between the P300 latency and severity of avoidance.

[58]	20 PTSD combat veterans 20 non-PTSD combat veterans	Target detection oddball, traumatic/neutral stimuli	Larger P300 amplitude in the PTSD group No difference inP300 amplitude between the target and non-target in the PTSD group; higher amplitude in response to the target stimuli in the control group. Earlier and 5 times greater P300 response to combat related pictures in PTSD patients. Repeated combat related pictures resulted in a rapid P300 amplitude reduction and latency prolongation. This effect was not observed for the target stimuli.

[59]	19 civil PTSD 17 subjects with numerous life events 18 without life events	Auditory oddball	Longer reaction times and lower amplitude P300 response

[60]	8 PTSD 8 non-PTSD combat veterans	Visual presentation of a sequence of trauma related and unrelated words	Newly identified P300tr component was suppressed to all stimuli in PTSD subjects

[43]	34 PTSD [25 male veterans/9 female victims of rape] 18 non-PTSD [10/8 from the same groups]	Auditory three-tone oddball	Smaller P300 response to the target tone. For women group, it was also smaller in response to the distracter tones.

[41]	9 PTSD [assault, rape, MVA, combat] 10 healthy controls	Modified stroop paradigm, visual presentation of neutral, positive and negative words	Smaller P300 amplitude to neutral, positive, and negative words in PTSD patients. Smaller response to neutral words as compared to positive and negative words.

[56]	16 medicated PTSD 9 un-medicated PTSD 10 healthy veterans	Auditory three-tone oddball	Significant decrease in P300 at Pz electrode in un-medicated PTSD group compared to the medicated PTSD and healthy subjects. Subjects with co-morbid panic disorder had the largest P300 amplitudes.

[39]	11 survivors of a ship fire with PTSD or near PTSD 9 controls with other psychiatric illnesses from the same ship	Auditory word and non-word oddball	Reduced P300 amplitude to non-words and negative words

[61]	25 combat veterans with PTSD/14 without PTSD	Auditory three-tone oddball	Reduced P300 amplitude to the target stimuli. Significant P300 amplitude enhancements at frontal sites to distracting stimuli during the novelty but not during the three-tone oddball tasks.

[62]	10 Vietnam war veterans with PTSD 10 without PTSD	Two oddball tasks of visual trauma-relevant and trauma-irrelevant threat (combat, social-threat, household, and neutral words)	Attenuated P300 response to neutral target stimuli Increased P300 amplitude in response to trauma-relevant combat stimuli but not to trauma-irrelevant social-threat stimuli at frontal electrode sites.

[28]	17 civil PTSD 17 healthy controls	Auditory oddball	Smaller P300 in PTSD, later at Pz

[32]	29 PTSD nurse veterans 38 non-PTSD	Three-tone oddball	Larger target P300 amplitudes in PTSD subjects

[38]	15 civil PTSD 15 controls	20 angry and 20 neutral faces	Slower P270 in the PTSD at occipital electrodes

[63]	25 combat PTSD 15 combat-exposed healthy controls	Three conditioned novelty visual and auditory oddball	No significant differences in P300 amplitude or latency regardless of stimulus type (target, novel) or modality (auditory, visual).

[35]	10 civil PTSD 10 controls	Auditory oddball	Same P3a amplitude in both groups, but there was a significant post-treatment attenuation of P3a in the PTSD group.

[64]	8 PTSD victims of Tokyo sarin attack 13 healthy controls	Auditory oddball	No difference in P300 latency. Significantly smaller P300 amplitudes in subjects with PTSD.

[65]	33 civil PTSD 33 matched controls	Auditory standard two-tone oddball	Delayed reduced P300 target amplitude, coupled with slower and less accurate target detection

[12]	10 male police/veteran PTSD 10 healthy controls	Auditory oddball	Smaller P550; More false negatives and positives; The higher the anxiety and depression level, the lower the amplitude; Reverse relationship between the P550 amplitude and intrusions.

[42]	19 PTSD 99 Alcohol dependence 16 personality disorder 25 anxiety or mood disorder	Visual presentation of happy, sad, and neutral faces	Longer P300 latency to happy stimuli in midline, central, and right frontal leads; Reduced P300 amplitude in response to neutral faces.

[40]	16 civil PTSD 15 trauma-exposed without PTSD 16 healthy controls	Modified auditory S1-S2 paradigm	Increased P300 and late positive complex amplitudes to trauma-specific questions; Only the PTSD group showed a differentiation between trauma-specific and neutral questions with respect to P300.

[66]	14 PTSD survivors of an air show disaster 15 trauma-exposed subjects without PTSD 15 healthy controls	Visual differential conditioning paradigm with traumatic/neutral pictures	Trauma-exposed subjects with and without PTSD showed successful differential conditioning to the trauma-relevant cue indicative of second-order conditioning

[57]	16 civil PTSD 16 schizophrenia 16 control subjects	Auditory oddball	Reduced amplitude of target and non-target P300 responses. Larger reduction in target P300 amplitude in left posterior parietal leads in PTSD group.

[49]	37 combat exposed veterans with PTSD and 47 without PTSD and their twins	Auditory oddball	No difference in P300 amplitude; When assessed the un-medicated nonsmoker group separately, P300 amplitude was smaller in the PTSD group

[1]	20 un-medicated and 14 medicated PTSD [mixed etiology] 136 controls	1-back working memory task	Reduced P300 working memory amplitude and delayed target P300 in PTSD. Amplitude reduction and delay of target P300 in medicated PTSD subjects. Little difference between the non-medicated PTSD subgroup and the controls.

[34]	12 PTSD and 12 control survivors of earthquake	Subliminal visual presentation of earthquake-related/unrelated words	Increased P2 and P3 amplitude in the PTSD group in response to the trauma-related stimuli

**Table 5:** Summary of studies with clinical correlates

Study	Clinical scales	Clinical correlates
[17]	IES, PTSD questionnaire	Positive correlation between P300 latency at Pz and Cz and the judged severity of intrusiveness measured by IES (Impact of Event Scale). Same applies to the level of intrusiveness measured by PTSD questionnaire. Negative correlation between P300 latency at Cz and severity of avoidance.

[52]	CAPS	P50 gating correlated negatively and significantly with PTSD subjects' CAPS re-experiencing intensity scores.

[56]	STAI	Measures of state anxiety (STAI) were significantly related to P300 amplitude at Pz; higher levels of self-reported state anxiety were associated with smaller P300 amplitudes.

[39]	CAPS	P300 amplitudes to emotionally meaningful words were significantly related to Clinician-Administered PTSD Scale subscales, in particular avoidance and arousal.

[25]	Mississippi score, STAI	Significant correlation between the magnitude of the MMN at Fz and the Mississippi PTSD Symptom Scale for civilian trauma.

[53]	CAPS, IES-R	No significant correlations were found between P50 gating and IES-R or CAPS total or subscale scores.

[28]	CAPS	Significant correlation between the intensity of numbing symptoms (reduced interest, social withdrawal, and emotional numbing) and P300 amplitude at parietal sites.

[30]	BDI, CAPS	PTSD subjects who showed N100 augmentation and P200 reduction were more depressed than PTSD patients with other patterns. Significant correlations between P200 slope and Clinician-Administered PTSD Scale total score, the Mississippi scale score, and the Hamilton depression score

[32]	Comorbidity CAPS, PCL-M	P2 slope was positively correlated with PCL-M, CAPS Total, and each of the CAPS subscale scores, indicating that a higher P2 slope was associated with more severe PTSD symptomatology P50 gating was negatively correlated with SCL-90-R Global Severity Index (GSI) score, indicating that worse gating is associated with more severe general psychopathology.

[63]	CAPS, IES-R, BDI, Mood State (POMS)	P300 amplitude to novel auditory stimuli increased as tension score in POMS increased.

[64]	CAPS, IES-R	Significantly negative correlation between present score of the cluster C of the CAPS (numbness/avoidance) and P300 amplitude at Pz.

[12]	STAI, BDI (Beck Depression Inventory), CAPS	Negative relationship between P550 amplitude and trait anxiety. Negative relationship between P550 amplitude and depression CAPS scores; negative relationship between P550 amplitude and intrusions.

[36]	Brief Symptom Inventory (BSI),	MMN was significantly correlated with the total PTSD score.

[1]	CAPS	Neither P300 nor behavioral measures were related to CAPS symptom severity measures.

## Results

Initial search yielded 57 papers of which 16 were solely EEG studies, another did not include a healthy control group, and three were children or adolescents studies, which were excluded from the current work. We excluded studies in children because of the small number of reports and a lack of homogeneity in ERP procedures used. Only a single study assessed CNV differences in PTSD patients [13] and thus it was not included in this review. After exclusion of these papers, 36 manuscripts had undergone comprehensive review. All of the presented data regarding the populations, their clinical characteristics (such as rating scales and scores when provided), and the utilized event-related response measures were collected from these 36 reports. Given the fact that most of the studies did not report possible comorbidities and medication regimens, these factors are not assessed or discussed in this review.

Of the 36 studies, 15 included veterans and/or military personnel with combat/war trauma and 17 included subjects exposed to civilian traumas such as motor vehicle accidents, rape and assault. Subjects of four studies were exposed to traumas of mixed etiology. All of the considered studies included healthy control subjects and most of them (especially combat related studies) included a third group of people who were exposed to trauma but did not develop PTSD based on the Diagnostic and Statistical Manual of Mental Disorders (DSM) III or IV diagnostic criteria.

A number of rating scales were used to assess PTSD symptomatology and comorbidities (Table 5). The most widely used scale was the Clinician-Administered PTSD Scale (CAPS), utilized in 10 of the 14 studies that evaluated clinical correlates. The CAPS is a well validated 'gold standard' 30-item structured interview that corresponds to the DSM-IV criteria for PTSD [14,15]. CAPS subscores include re-experience (intrusiveness), avoidance and arousal. Other assessment instruments included the Mississippi scale for PTSD [16] (one study), a PTSD questionnaire [17] (one study), State-Trait Anxiety Inventory (STAI) [18] (three studies), brief symptom inventory [19,20] (one study), Beck depression inventory [21] (two studies), Impact of Events Scale-Revised [22] (IES-R) (four studies), PTSD Check List-Military (PCL-M) [23] (one study), and Profile of Mood States (POMS) [24] (one study).

Standard tone sounds were used for the auditory oddball and gating studies. For the visual event-related potential paradigms, mostly three sets of images were presented to the subjects. These included neutral images such as nature scenes, trauma-specific aversive images and non-trauma-specific aversive pictures. Auditory stimuli were used in 22 studies, 13 used visual stimuli, and one study included both auditory and visual stimuli in their paradigms.

Most of the studies focused on the ERP component P300 (26 studies). P50, N100 and P200 components were also assessed by a fair number of studies (9 studies P50; 13 studies N100; and 10 studies P200). P100 (visual), N200 and the MMN were the least studied ERP components. Many studies probed more than one ERP component; this explains why the sum of the number of entries in tables 1, 2, 3 and 4 is larger than the total number of papers reviewed. The ERP components that were considered by at least four studies are summarized in the tables.

We first grouped all the studies together for each ERP component and counted the number of studies which reported differences in the amplitudes in each direction. We then separated studies in auditory/visual modalities and amplitudes reported in response to the trauma related (TR) and trauma nonrelated (TNR) stimuli when applicable.

### P50 amplitude and gating (Table 1)

Nine papers examined the P50 auditory event-related response. Two papers only examined the amplitude and latencies of the P50 components (in other words, not using paired stimuli) of which one showed an exaggerated response to the tone and one did not [25]. Seven papers utilized the standard paired-stimulus paradigm to examine sensory gating. Six papers examining habituation showed significantly elevated gating ratios (in other words, the decreased ability to inhibit or suppress repeating (redundant) incoming sensory input) in PTSD patients as compared to controls. One study found impaired P50 gating only in the right hemisphere in PTSD patients as compared to the control group [26]. In regards to S1, four of the seven papers did not reveal a difference in the amplitude of response to S1 while one found a reduced response to S1 paired with an exaggerated response to S2 in PTSD subject [27]. Hunter and colleague's study [26] found reduced S1 strength only in the right hemisphere of patients with PTSD. To summarize, a small number of studies evaluated the P50 component in PTSD and among these, the majority reported impaired gating of the repetitive stimuli.

### N100 (Table 2)

Thirteen studies assessed the N100 midlatency auditory event-related response. Six studies reported increased N100 amplitude in subjects with PTSD, two reported a reduction in N100 amplitude in this population, and four did not find a difference. One study did not report the N100 amplitudes. Of the nine studies reporting latencies, four found increased N100 latencies in PTSD patients and five did not find a difference in latency between the PTSD subjects and healthy controls. From seven auditory oddball studies, one did not report the amplitudes, four reported no differences, and two reported increased amplitude in the N100 component in the PTSD patients. Two studies presented emotional faces in the visual modality, one of which reported increased amplitude and the other reported reduced amplitude to sad faces. In summary, N100 ERP component findings are overall inconsistent in existing PTSD studies.

### P200 (Table 3)

Ten studies examined the P200 MLAER component. Three studies found decreased P200 amplitude in PTSD patients [25,28,29]. In two studies, a linear relationship was found between the tone intensity and P200 amplitude in the control group, which was not detected in combat PTSD patients [30,31]. In other words, in contrast to the control subjects, PTSD patients failed to show increased P200 amplitude in response to increased tone intensity. In contrast, Metzger and colleagues reported increased P200 amplitude and intensity slope in PTSD patients [32,33]. In one of these studies, the slope was correlated with re-experience symptoms cluster but not the other symptom cluster scores. Yun and colleagues [34] found increased P2 amplitude in response to subliminal visual presentation of TR stimuli in PTSD patients. Two studies failed to detect a difference between the PTSD patients and healthy controls in P200 amplitudes or latencies [35,36]. Wessa and colleagues [29] reported reduced P200 amplitude with lack of differentiation between the TR and TNR visual stimuli in the PTSD patients.

To sum, from ten studies that compared P200 amplitudes between PTSD patients and controls, one reported increased amplitude in PTSD patients (in response to subliminal presentation of the stimuli), three reported reduced amplitude and two reported increased amplitude/slope intensity. Two studies failed to show increased P200 amplitude in response to increased intensity in PTSD patients, and two did not detect any difference between the PTSD and control groups. Given the close proximity of the N100 and P200 components, we examined the difference in the amplitudes of these two components in PTSD studies. Among 13 studies reporting N100 amplitudes, six reported an increase while none of the five P200 studies which reported the amplitude of this component found increased amplitudes. Utilizing Fisher's exact test of 2 × 2 contingency tables, a trend towards difference in the behavior of the two components was detected (*P *(2-tails) = 0.102, power (2-tails) = 0.503) [37].

### P300 (Table 4)

Most of the studies examining event-related responses in PTSD populations (26 studies) assessed P300 component in either an auditory or a visual oddball design.

#### Auditory studies

Seventeen studies assessed the P300 component in an auditory modality, 15 of which were standard oddballs. One of these 15 studies did not report the P300 amplitude [38]. Eleven studies reported reduced response amplitude to the target stimuli, one reported increased amplitude, and two failed to detect any difference between the PTSD and the control subjects. One of these studies [39] reported reduced amplitude to non-word stimuli (reversed waveforms of the digitized words) and increased amplitude to positive words in PTSD patients. Although Metzger and colleagues [33] did not find a difference in P300 amplitude, when they evaluated a subgroup of nonmedicated, nonsmoker subjects, again they reported decreased P300 amplitude in PTSD subjects. Wessa and colleagues [40] showed increased response amplitude to TR questions.

#### Visual studies

Eight studies reported P300 responses to TR versus aversive TNR or neutral stimuli. One study [34] reported an increased P300 response to the subliminal presentation of the TR stimuli in PTSD patients. Due to different methodology, this study is not included in the following analysis. Five studies reported increased amplitude to the TR stimuli in PTSD patients compared to the controls and one study reported increased response to all stimuli in PTSD patients. Although Metzger and colleagues [41] reported smaller P300 amplitude to all the stimuli in the PTSD patients, they found a smaller response to the neutral words as compared to the positive and negative words. Ehlers and colleagues [42] reported reduced amplitude to neutral faces in the PTSD patients and Veltmeyer and colleagues [1] in an N-back working memory task found reduced P300 amplitude in the PTSD group.

We also reviewed P300 responses to TR/aversive stimuli and neutral stimuli separately irrespective of the modality of stimulus presentation (auditory or visual). From the 17 reports on the P300 amplitudes in response to the visual or auditory TNR/neutral stimuli, 13 studies reported reduced amplitude in PTSD patients, two reported increased amplitude, and two studies failed to detect any difference between the PTSD subjects and the control group.

Eight studies reported P300 responses to TR/aversive stimuli. Seven studies found increased P300 amplitude in the PTSD patients and one study reported reduced response in the PTSD patients as compared to the control group [43]. In this last study, within group comparison showed a smaller response to the neutral words as compared to the positive and negative words in PTSD patients. On aggregate (studies pooled across visual or auditory modalities), seven out of eight studies reporting P300 responses to TR reported increased amplitudes while only two of sixteen studies examining responses to TNR stimuli reported increased P300 amplitudes (Fisher's exact test of 2 × 2 contingency tables, *P *(2-tailed) < 0.001, power (2-tailed) = 0.986). Conversely, while 13 of 17 papers reported decreased P300 amplitudes to TNR stimuli, only one of eight papers reported decreased amplitudes to TR stimuli (Fisher's exact test of 2 × 2 contingency tables *P *(2-tailed) < 0.008, power (2-tailed) = 0.889).

In summary, the majority of studies on P300 component reported sensitization of the P300 response to TR stimuli and dampening of this response to the neutral stimuli.

### Other event-related responses

A host of studies examined other event-related responses including visual P100, N200 (a negativity generated with stimulus deviation), MMN (also a negativity detected with stimulus deviation) and the late positive auditory complexes. Wessa *et al*. [29] reported a later positivity (P550) and skin conductance to be negatively correlated with the severity of avoidance symptoms. None of these smaller bodies of literature included more than three published, full length papers and none has shown a consistent pattern. We concluded that these smaller bodies of literature were not yet at a stage that would significantly contribute to our understanding of PTSD and did not include them in this analysis.

Of all the studies reviewed, 14 examined the correlations between the assessed ERP measures and PTSD symptomatology. While 17 significant correlations were reported (see Table 5), a large number of trend level or non-significant correlations are also reported. Due to different methods and clinical rating scales used among the studies, we could not make a conclusion about the clinical correlates of the reported ERP components.

## Discussion

In this manuscript, we reviewed reports that assessed ERP components in subjects with PTSD and healthy subjects. The early stage of IP was not examined due to a paucity of studies. Most of the studies presented evidence for a difference in IP between patients with PTSD and control patients during the entire midlatency range and extending into the later stages of IP.

The earlier part of the midlatency range is almost entirely pre-attentive and reflects the automatic processes of stimulus registration (reflected by response amplitudes) and filtering processes (reflected by sensory gating measures). Processes occurring at this stage are likely substantially bottom-up in nature and might be clinically correlated with 'intrusiveness' and 'hyperarousal', as these symptoms are likely to be automatic and, to a degree, pre-attentive. On the other hand, the attentive top-down level of IP is usually probed by examining the later occurring ERPs like the P300 or P550. This level of information processing could be clinically related with volitional 'avoidance' symptoms.

Results of reviewed P50 gating studies show some evidence of impaired pre-attentive habituation in PTSD patients. Whether gating deficit results from exposure to stress or represents a pre-existing vulnerability to developing PTSD can only be determined through the conduct of careful longitudinal studies. Current literature shows that decreased gating is not uncommon in seemingly healthy individuals [44,45]. On the other hand, studies also show that laboratory-induced stress (which, by definition, cannot be severe or chronic) can decrease gating in healthy individuals with normal baseline gating [46]. It is thus quite plausible to postulate that severe or chronic stress can be detrimental to the sensory gating function and possibly individuals with premorbid deficient sensory gating function are more susceptible to developing PTSD when subjected to this form of stress.

The noted possibility that the N100 and P200 components may be affected differently in association with PTSD is worthy of further investigation as the P200 along with the N100 form the vertex complex and are considered closely related. While experimentally dissociable, the two components share similar neural sources and topographical distributions [47]. A significant variance in the effects of stimulation on these two components would indeed be an interesting finding worthy of further exploration, as it could yield clues to the nature and timing of IP difficulties in this patient population. In fact, despite the small number of studies, a trend suggesting that the two components behave differently in association with PTSD is seen.

The most widely examined ERP component is the P300, which reflects task allocation of cognitive resources [3]. In a first glance at the reports that evaluated the P300 component, it is difficult to draw a conclusion as the results are contradictory. When divided into two groups of studies with presentation of visual and auditory stimuli, a higher number of auditory studies show reduced P300 amplitude while a larger number of visual studies show increased P300 amplitude in the PTSD patients. This could be due to the fact that most of the auditory studies are standard oddballs with presentation of neutral stimuli while a larger number of studies in the visual modality presented TR/aversive images as non-target stimuli. In other words, when results are examined in terms of relevance of the stimuli to the trauma, they appear to be more meaningful. Among 18 studies that reported P300 amplitude in response to the TNR/neutral stimuli in auditory/visual modalities, the majority - 13 studies - showed reduced amplitude while only two of the studies showed increased amplitude. Furthermore, among the eight studies which evaluated P300 responses to the TR/aversive stimuli, seven studies found increased P300 amplitude in patients with PTSD when compared to the control group. Of interest, the one study which showed reduced P300 amplitude in PTSD patients, in within group comparison, showed a smaller response to the neutral words as compared to the positive and negative words in PTSD patients. These findings suggest the existence of two possible P300-related abnormalities: a significant decrease in responses to TNR stimuli and a significant increase in TR stimuli. Whether these two abnormalities occur simultaneously or sequentially and whether these two abnormalities reflect different aspects of the PTSD syndrome are currently open questions. Based on the above, there might be an increased attentional resource allocation in subjects with PTSD to the cues of trauma at the expense of the neutral stimuli. In other words, subjects with PTSD show sensitization and impaired habituation to the stimuli which represent the traumatic stimuli. Considering the limited attentional resources (which might be even more limited in patients with PTSD due to causes such as traumatic brain injury), this shift in allocation of these resources (sensitization) takes place at the expense of reduced response to and processing of neutral stimuli. In a clinical language, this attentional phenomenon may be translated into hyperarousal to any reminder of the traumatic event at expense of the neutral and nonaversive stimuli. In a further speculative step, this sensitization to the TR cues/stimuli, may be extended to the top-down filtering of the TR memories and assumed to be responsible for impaired inhibition of internal TR stimuli/memories which present in the form of re-experiences, intrusive memories and flash backs.

Whether ERP findings in individuals with PTSD are the result of trauma, or are present in patients who are more susceptible to PTSD when subjected to a traumatic experience remains an open question. Hypersensitivity to aversive stimuli and lack of habituation to them could be a premorbid difference in people who are more susceptible to developing PTSD after a traumatic experience. This possibility becomes more important in light of the fact that most ERP components are heavily genetically influenced [48]. Whether the ERP findings are characteristic of people susceptible to PTSD or are results of the trauma needs to be further assessed in longitudinal cohort studies (for example, pre- and postdeployment studies in veterans). Twin studies may be utilized in assessing whether the different findings in subjects are inborn genetic characteristics or a result of traumatic experience. This review found only two twin studies, both of which reported differences in P200 and P300 components between the PTSD subjects and their non-PTSD identical twin [49,33]. Although these results suggest that the information processing differences are acquired, more studies are needed to support these findings.

Tables 1, 2, 3 and 4 show that, although the amplitudes of the ERP components are examined in most of the reviewed studies, literature is very sparse in terms of the latency of these components. A negative correlation between the latency of the P300 and severity of avoidance has been found [50], in other words, the stronger the avoidance, the shorter the P300 latency. As latency of a component reflects the number of synaptic links leading to the development of the response [3], this observation might suggest that an altered process might be engaged in some patients with PTSD. Furthermore, the same study presents a positive correlation between latency and severity of the intrusiveness symptoms. Increased latency might thus suggest altered IP pathways in PTSD and support the need for close examination of latencies in ERP-PTSD studies. Differences in the direction of correlation between avoidance and latency, and intrusiveness and latency in the same patient population may point at different pathways being involved in the formation of different symptom clusters in PTSD. While highly speculative (based on a single report) these findings are of significant interest and deserve further exploration.

Given the large number of ERP variables examined as well as the large number of PTSD symptoms (and the varied PTSD assessment methods) no clear trends can be discerned (Table 5). If ERP components indeed reflect specific symptom type or severity, this can potentially be useful in both diagnostic evaluation and treatment monitoring. However, in those studies in which clinical correlations were assessed, comparable rating scales were not utilized. As a result, for the purpose of future ERP-PTSD studies, scales providing detailed accounts of symptom severity would be most suitable to examine any clinical-electrophysiological correlations. More importantly, it will be vital to analyze and include symptom-cluster information in the reports given the current debate over the make-up of symptom composition of the PTSD clusters. Whether ERP methodologies and measures can be used to guide the development of new or revised, empirically-based symptom clusters might also be a fruitful consideration in future studies.

## Conclusions

In summary, our review points to a potential relationship between ERP measures and PTSD. The most consistent findings include diminished habituation to repetitive stimuli as evidenced by reduced P50 gating, and sensitization of the P300 response to TR stimuli. The P300 response to neutral stimuli, on the other hand, is diminished in PTSD patients, suggesting a potential 'trade off' between processing traumatic versus neutral stimuli. These differences in IP are consistent with the findings in emotional processing studies in PTSD, which reveal increased emotional response to the cues of trauma. Corresponding differences in IP and emotion responses can help in a more comprehensive understanding of PTSD. Although emotion regulation and processing is more extensively addressed in research, early cognitive processing of the information needs further exploration for a better understanding of the whole picture. The findings of this review may also translate to better understanding of the mechanisms involved in clinical symptoms development. Results on the N100 and P200 components are not as conclusive and other components are not addressed in the majority of the studies.

The inconsistency in the results of different studies can stem from differences in methodologies and patient populations or differences in illness-related variables. Other contributing factors can be physiological or external factors such as patients' personality traits, pre-existing factors (such as comorbid illnesses, presence of alcohol or drugs and tiredness) or contributing conditions (availability or lack of support, use of medications such as morphine, beta blockers, and benzodiazepines) prior to or immediately after the traumatic experience. Unfortunately, many of the reviewed literature failed to assess or report comorbid illnesses or medication regimens. Prospective investigations with more uniform methodologies, unified patient populations, and pre- and post-trauma approaches are necessary to further explore such relationships.

## Competing interests

The authors declare that they have no competing interests.

## Authors' contributions

AJ participated in design of the study, data collection and analysis; interpretation of the results and conceptualization of the findings; and preparation of the manuscript. IL participated in data analysis; interpretation of the results and conceptualization of the findings; and preparation of the manuscript. AA participated in design of the study; interpretation of the results and conceptualization of the findings; and preparation of the manuscript. KG participated in data analysis, and preparation of the manuscript.

NNB participated in design of the study; data analysis, interpretation of the results and conceptualization of the findings; and preparation of the manuscript. All authors read and approved the final manuscript.
